# Associations of plasma hepcidin with mortality risk in patients with coronary artery disease

**DOI:** 10.18632/oncotarget.22722

**Published:** 2017-11-27

**Authors:** Xinrui Li, Ding Ding, Yuan Zhang, Dongfang Su, Min Wang, Xuechen Chen, Yan Yang, Changjiang Hong, Gang Hu, Wenhua Ling

**Affiliations:** ^1^ Guangdong Provincial Key Laboratory of Food, Nutrition and Health, Department of Nutrition, School of Public Health, Sun Yat-sen University, Guangzhou, Guangdong, China; ^2^ Department of Cardiology, General Hospital of Guangzhou Military Command of People’s Liberation Army, Guangzhou, Guangdong, China; ^3^ Chronic Disease Epidemiology Laboratory, Pennington Biomedical Research Center, Baton Rouge, LA, USA

**Keywords:** hepcidin, coronary artery disease, mortality, cohort study

## Abstract

**Background:**

Increased blood hepcidin may be associated with the presence and promotion of atherosclerosis, the association of hepcidin with mortality among coronary artery disease (CAD) patients remains unknown. We sought to assess the relationship of hepcidin and all-cause and cardiovascular disease (CVD) mortality among CAD patients with and without acute coronary syndrome (ACS).

**Methods and Results:**

This study included 759 patients with ACS and 526 patients with stable CAD. After an average follow-up of 4.1 years, 154 deaths were recorded, 114 were due to CVD. After adjusting for CVD risk factors and inflammatory markers, the plasma hepcidin was positively associated with all-cause and CVD mortality in the ACS patients, the multivariable-adjusted hazard ratios (HRs) across tertiles of hepcidin were 1.00, 2.18 (95% CI 1.23-3.94), and 2.82 (95% CI 1.59-5.12) for all-cause mortality (*P*_trend_=0.006), and 1.00, 2.20 (95% CI 1.12-4.05), and 2.64 (95% CI 1.41-5.65) for CVD mortality (*P*_trend_=0.01). The C-index and net reclassification improvement when including hepcidin in traditional CVD models were 1.6% and 21.5% for all-cause mortality, 1.4% and 23.5% for CVD mortality, respectively, (*P*<0.001).

**Conclusions:**

Plasma hepcidin was positively associated with mortality in ACS patients. Hepcidin may be a potential biomarker for risk prediction in ACS patients.

## INTRODUCTION

Cardiovascular disease (CVD) is a leading cause of morbidity and mortality world-wide, with acute coronary syndrome (ACS) being the most common initial presentation [1]. ACS is usually the result of an atherosclerotic plaque rupture. Coronary plaques vulnerable to rupture are characterized by a lipid-laden necrotic core in which macrophage infiltration leads to the release of inflammatory cytokines [2]. This initiates a vicious inflammatory process, which involves thinning of the fibrous cap, typically around the “shoulders” of the plaque [3].

Many population studies have revealed that some biomarkers were specific to ACS or stable plaques, since circulating levels of macrophage activity (neopterin) and tissue remodeling (matrix metalloproteinase-9) were consistently higher among ACS patients compared to stable patients [4, 5]. Improvements in the diagnosis of ACS through more sensitive biomarkers of ACS has partly led to the reduction in the rates of myocardial infarction (MI) over the last decade with concomitant improvements in patient survival [6]. However, clarification of new biomarkers that may be different specific for the prognosis of ACS and stable coronary artery disease (CAD) needs further clinical investigation.

The iron hypothesis, which states that excessive iron would contribute to the pathogenesis of CAD, was first proposed by Sullivan in 1981 [7]. However, subsequent epidemiological studies about the association of circulating iron, non-iron binding capacity, transferrin receptor, and ferritin with atherosclerosis have shown conflicting results, with some studies confirming [8, 9] and others denying this possible deleterious effect [10, 11]. Hepcidin has been recently recognized as the major regulatory protein in iron metabolism. This peptide hormone plays an important role in systemic regulation of iron homeostasis [12]. It has been hypothesized that hepcidin may slow or prevent the mobilization of iron from macrophages and lead to an increased cardiovascular disease risk, such as atherosclerosis and other cardiovascular disease such as Kawasaki disease [13–15]. In experimental settings, increased hepcidin expression has been found in both non-ischemic and ischemic myocardium after MI [16, 17]. In epidemiological studies, higher hepcidin concentrations have been shown to be related to metabolic syndrome, as well as to the presence of plaque and intima-media thickness by non-invasive measurements of atherosclerosis in women [18, 19]. In addition, animal studies have shown that hepcidin promoted plaque destabilization partly by exaggerating inflammatory cytokine release, intracellular lipid accumulation, oxidative stress, and apoptosis in the macrophages with iron retention [20]. However, the association of circulating hepcidin levels and mortality risk in CAD patients, especially ACS and stable patients separately remains unknown.

The aim of this study was to determine plasma hepcidin levels in patients under ACS or stable CAD condition and to assess the association of hepcidin with the risks of all-cause and CVD mortality among ACS and stable CAD patients.

## RESULTS

The baseline characteristics according to type of CAD among the 1285 CAD patients reported in Table 1. ACS patients were similar to stable CAD patients with respect to BMI, duration of CAD, blood pressure and lipids; there were significantly more males, smokers, diabetes patients, higher C-reactive protein (CRP) and IL-6 levels, and more severe conditions in the degree of coronary angiography (CAG) degree in the ACS patients.

**Table 1:** Baseline characteristics of patients with CAD

Baseline characteristics	Total	Acute coronary syndrome	Stable CAD	*P* value
Patients, n	1285	759	526	
Age, y	64.8 (10.8)	64.1 (11.2)	65.8 (10.2)	0.05
Female, %	33.5	29.0	39.9	<0.001
Body mass index, kg/m^2^	23.9 (3.32)	23.8 (3.32)	24.0 (3.32)	0.33
Systolic blood pressure, mm Hg	134 (23)	134 (23)	136 (23)	0.38
Diastolic blood pressure, mm Hg	77 (13)	76 (13)	77 (12)	0.54
Triglyceride, mmol/L	1.85 (1.28)	1.87 (1.20)	1.82 (1.38)	0.66
Low-density lipoprotein cholesterol, mmol/L	2.98 (0.98)	3.02 (1.01)	2.94 (0.94)	0.06
Hemoglobin, g/L	130(20.4)	130(21.6)	129(18.3)	0.83
Iron, μmol/L	13.0 (6.5)	12.6 (6.7)	13.7 (6.2)	<0.001
Unsaturated iron-binding capacity, μmol/L	28.2 (12.8)	28.3 (14.0)	28.2 (10.7)	0.84
Transferrin saturation, %	33.5 (15.7)	32.8 (15.9)	34.4 (15.4)	0.08
Soluble transferrin receptor, mg/L	3.18 (2.04)	3.15 (2.21)	3.21 (1.77)	0.80
C-reactive protein, mg/L	3.80 (1.00-13.9)	6.50 (1.55-18.8)	1.92 (0.72-6.16)	<0.001
Interleukin-6, pg/mL	1.64 (0.66-3.50)	1.78 (0.80-4.01)	1.48 (0.56-2.96)	<0.001
Ferritin, ng/mL	285 (168-436)	317 (198-486)	229 (146-367)	<0.001
Hepcidin, ng/mL	24.6(14.1-39.7)	27.1(17.2-42.6)	21.3 (11.2-34.5)	<0.001
Duration of CAD, y				
First diagnosed CAD (n=753)	--	--	--	--
History of CAD (n=532)	3.80 (1.17-9.01)	3.44 (1.00-8.79)	4.00 (1.44-9.49)	0.08
Years of education, %				0.77
≤9	60.5	61.3	59.2	
10-12	21.6	20.9	22.7	
≥13	17.9	17.8	18.1	
Leisure-time physical activities, %				0.09
None	32.3	34.9	28.5	
<30 minutes/day	22.2	20.8	24.2	
≥30 minutes/day	45.5	44.3	47.3	
Smoking, %				<0.001
Never	60.5	55.7	67.5	
Past	9.9	8.5	11.9	
Current	29.6	35.8	20.6	
Alcohol use, %				0.62
Never	77.7	78.4	76.8	
Past	7.6	7.0	8.6	
Current	14.7	14.6	14.7	
Coronary artery stenosis degree of Coronary Angiography, %				<0.001
50-74.9%	24.3	14.0	47.5	
≥75%	75.7	86.0	52.5	
Prevalence of hypertension, %	92.6	93.8	90.9	0.05
Prevalence of diabetes, %	43.1	46.8	37.9	0.002
Uses of medications, %				
Antiplatelet/anticoagulant medication, %	88.2	90.6	84.6	0.001
Antihypertensive medication, %	87.9	89.3	85.9	0.06
Lipid-lowering medication, %	81.9	84.8	77.6	0.001
Glucose-lowering medication, %	25.9	26.7	24.7	0.26

Continuous data are reported as M (SD) if normally distributed and median (25^th^, 75^th^ percentile) if non-normally distributed, categorical data are reported as percentages. All continuous variables are adjusted for age and gender, except for age (adjusted for gender only).

CAD, coronary artery disease.

**Figure 1:**
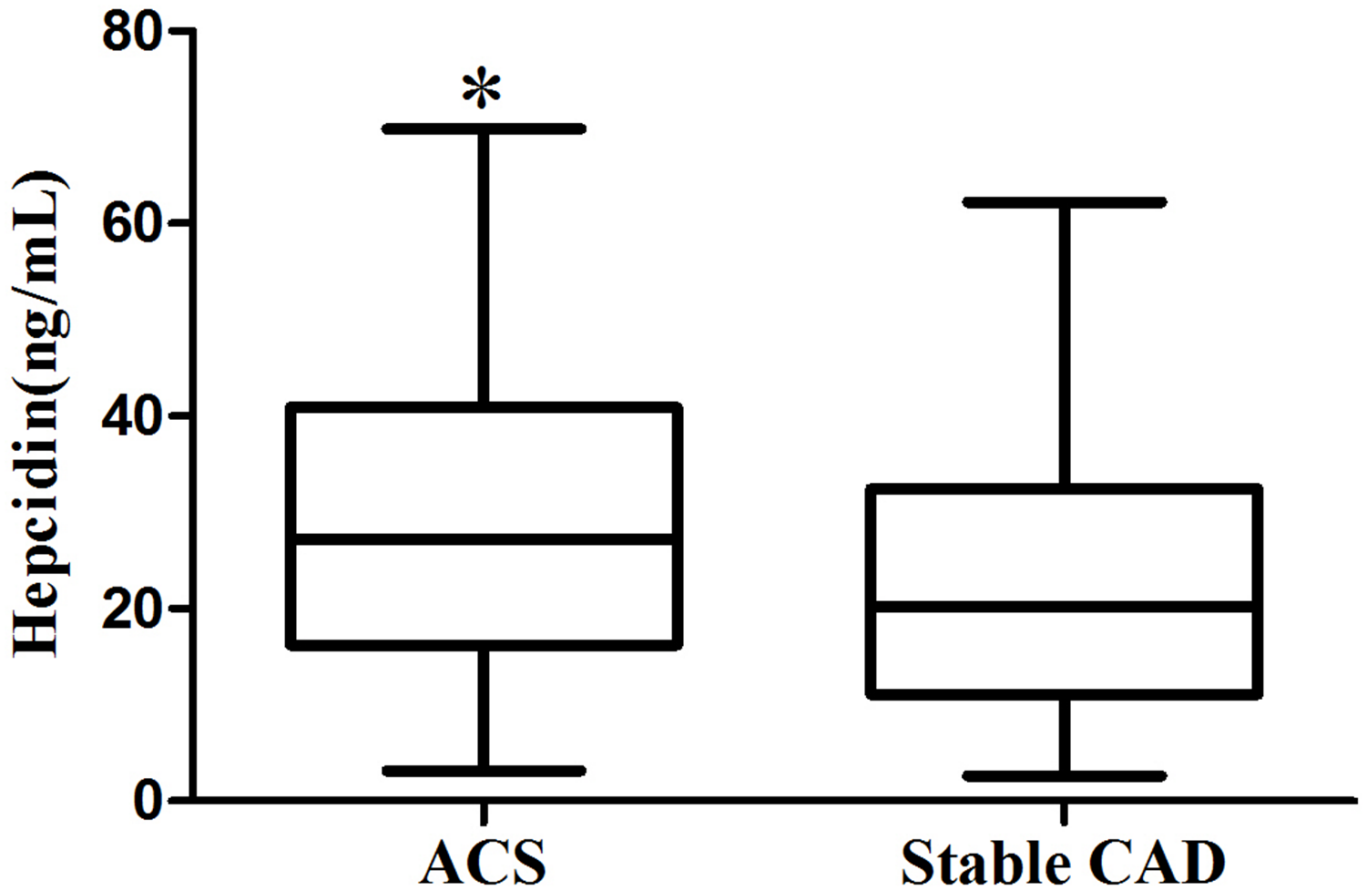
Whisker plot showing plasma hepcidin levels in ACS and stable CAD patients In these plots, middle bars represent the median values; the upper and lower bars represent the 25^th^ and 75^th^ percentiles, respectively.

Plasma hepcidin level was higher in patients with ACS than in patients with stable CAD (Figure 1). Hepcidin was strongly correlated with ferritin in both ACS (r=0.845, *P*<0.05) and stable CAD patients (r=0.831, *P*<0.05). Ferritin and hepcidin were positively related with CRP or IL-6 among both ACS and stable patients (Supplementary Table 1).

During a median follow-up of 4.1 years, 154 deaths were recorded (87 in ACS and 67 in the stable CAD groups), 114 of which were due to CVD (66 in ACS and 48 in stable CAD groups). Because the interactions between gender and hepcidin on the risks of all-cause and CVD mortality were not statistically significant, data for men and women were combined in the analyses to maximize the statistical power. Among CAD patients, hepcidin levels were positively associated with all-cause and CVD mortality, although this trend was not statistically significant after adjusting for CRP and ferritin (Table 2).

**Table 2:** Hazard ratios for all-cause and cardiovascular mortality according to different levels of hepcidin among patients

	Baseline plasma hepcidin level			*P*_*trend*_	as a continuous variable^e^
	<33.3%	33.3 to <66.7%	≥66.7		
**Total samples**					
No. of subjects	428	429	428		1285
Person-years	1788	1763	1621		5172
All-cause mortality					
No. of death	41	49	64		154
Adjustment for age and gender	1.00	1.21 (0.81-1.48)	1.81 (1.35-2.37)	0.02	1.65 (1.05-2.54)
Multivariable adjustment					
Model 1^a^	1.00	1.15 (0.69-1.71)	1.80 (1.23-2.45)	0.03	1.90 (1.25-3.01)
Model 2^b^	1.00	1.13 (0.65-1.70)	1.75 (1.21-2.49)	0.04	1.83 (1.21-2.96)
Model 3^c^	1.00	1.06 (0.62-1.64)	1.65 (1.11-2.33)	0.10	1.59 (1.12-2.59)
Model 4^d^	1.00	1.03 (0.59-1.63)	1.49 (1.05 -2.21)	0.15	1.41 (0.95-2.29)
Cardiovascular mortality					
No. of death	33	35	46		114
Adjustment for age and gender	1.00	1.12 (0.71-1.65)	1.74 (1.15-2.71)	0.01	2.01 (1.35-3.25)
Multivariable adjustment					
Model 1^a^	1.00	1.11 (0.69-1.80)	1.91 (1.15-2.80)	0.01	2.15 (1.45-3.34)
Model 2^b^	1.00	1.09 (0.68-1.76)	1.83 (1.13-2.79)	0.02	2.05 (1.25-3.24)
Model 3^c^	1.00	1.03 (0.59-1.61)	1.65 (1.06-2.63)	0.06	1.89 (1.17-2.92)
Model 4^d^	1.00	0.85 (0.69-1.53)	1.39 (0.79-2.41)	0.15	1.52 (0.87-2.50)
**Acute coronary syndrome**					
No. of subjects	252	254	253		759
Person-years	1064	1008	918		2990
All-cause mortality					
No. of death	17	30	40		87
Adjustment for age and gender	1.00	1.92 (0.85-3.56)	3.41 (1.81-6.05)	<0.001	3.33 (1.82-5.45)
Multivariable adjustment					
Model 1^a^	1.00	2.10 (1.05-3.59)	3.31 (1.79-5.94)	<0.001	3.28 (1.75-5.63)
Model 2^b^	1.00	2.32 (1.31-4.25)	2.97 (1.68-5.45)	<0.001	3.15 (1.43-5.60)
Model 3^c^	1.00	2.18 (1.23-3.94)	2.82(1.59-5.12)	0.006	2.85 (1.40-5.21)
Model 4^d^	1.00	2.15 (1.11-3.86)	2.65 (1.37-4.85)	0.003	2.76 (1.35-4.87)
Cardiovascular mortality					
No. of death	13	21	32		66
Adjustment for age and gender	1.00	2.36 (0.85-4.22)	3.23 (1.51-5.92)	<0.001	3.43 (1.35-5.78)
Multivariable adjustment					
Model 1^a^	1.00	2.22 (1.23-4.15)	3.18 (1.60-5.88)	<0.001	3.35 (1.63-6.25)
Model 2^b^	1.00	2.18 (1.15-3.98)	2.94 (1.57-5.75)	0.009	3.30 (1.70-7.12)
Model 3^c^	1.00	2.20 (1.12-4.05)	2.64 (1.41-5.65)	0.01	3.12 (1.52-6.43)
Model 4^d^	1.00	2.15 (1.08-3.87)	2.48 (1.31-5.05)	0.03	2.75 (1.39-6.12)
**Stable coronary artery disease**					
No. of subjects	175	176	175		526
Person-years	723	739	719		2181
All-cause mortality					
No. of death	23	22	22		67
Adjustment for age and gender	1.00	0.92 (0.72-1.78)	1.03 (0.66-1.79)	0.75	1.10 (0.72-2.05)
Multivariable adjustment					
Model 1^a^	1.00	0.95 (0.65-1.66)	0.90 (0.63-1.78)	0.88	0.98 (0.66-1.99)
Model 2^b^	1.00	0.88 (0.62-1.73)	1.01 (0.55-2.01)	0.76	1.05 (0.55-2.32)
Model 3^c^	1.00	0.76 (0.56-1.66)	0.92 (0.60-1.82)	0.68	0.95 (0.53-2.05)
Model 4^d^	1.00	0.80 (0.52-1.63)	0.73 (0.45-1.65)	0.72	0.88 (0.40-1.66)
Cardiovascular mortality					
No. of death	15	15	18		48
Adjustment for age and gender	1.00	1.10 (0.60-1.82)	1.15 (0.55-1.92)	0.97	1.03 (0.56-2.31)
Multivariable adjustment					
Model 1^a^	1.00	1.00 (0.58-2.03)	1.03 (0.54-1.88)	0.88	0.89 (0.54-2.11)
Model 2^b^	1.00	1.03 (0.53-2.12)	0.98 (0.52-1.95)	0.95	0.95(0.49-1.98)
Model 3^c^	1.00	0.94 (0.48-1.98)	0.88 (0.63-2.12)	0.79	0.99 (0.46-2.05)
Model 4^d^	1.00	0.82 (0.50-1.73)	0.72 (0.56-1.87)	0.76	0.78 (0.33-1.89)

^a^model 1 was adjusted for age, gender, smoke and alcohol use.

^b^Model 2 was further adjusted for body mass index, systolic blood pressure, low-density lipoprotein cholesterol, history of diabetes, history of heart failure, type, severity, and treatment of coronary artery disease, use of lipid-lowering, anti-hypertensive and glucose-lowering drugs.

^c^Model 3 additionally adjusted for C-reactive protein.

^d^Model 4 additionally adjusted for ferritin.

^e^Hepcidin was log-transformed when included in the models as a continuous variable.

There was significant interaction between the type of CAD and plasma hepcidin on the risks of all-cause and CVD mortality (*P*_interatcion_<0.05). After stratification by type of CAD, hepcidin was positively associated with all-cause and CVD mortality among ACS patients. The multivariable-adjusted hazard ratios (HRs) across three hepcidin categories were 1.00, 2.18 (95% CI 1.23-3.94), and 2.82 (95% CI 1.59-5.12) for all-cause mortality (*P*_trend_=0.006), and 1.00, 2.20 (95% CI 1.12-4.05), and 2.64 (95% CI 1.41-5.65) for CVD mortality (*P*_trend_=0.01). After further adjustment for plasma ferritin levels, hepcidin remained related to risks of all-cause and CVD mortality with this significantly positive association (Table 2). However, there was no statistically significant association between hepcidin and mortality in the stable CAD group (Table 2).

The C index was used to assess and quantify the improvement in risk prediction for all-cause and CVD mortality offered by hepcidin. The differences in the C index, when including or excluding hepcidin, were 1.6% (0.785 vs. 0.769, *P*<0.001) for all-cause mortality and 1.4% (0.793 vs. 0.779, *P*<0.001) for CVD mortality in ACS patients. When we added hepcidin to the clinical variables to predict all-cause and CVD mortality risk for ACS patients, 42.5% and 40.9% of dead patients were correctly reclassified to a higher risk category, respectively, and 11.4% and 10.6% incorrectly reclassified to lower risk category, respectively. Similarly, for all-cause and CVD mortality risk for ACS patients, 7.14% and 7.34% of surviving patients were correctly reclassified to a lower risk category, respectively, and 16.7% and 14.1% incorrectly reclassified to higher risk category, respectively. So the estimated Net reclassification improvement (NRI) was 21.5% (95% CI 10.5-35.7%, *P*<0.001) for all-cause mortality and 23.5% (95% CI 9.51-38.4%, *P*<0.001) for CVD mortality by including hepcidin (Table 3).

**Table 3:** Reclassification of predicted risk with the addition of hepcidin in acute coronary syndrome patients

Predicted risk (without hepcidin)	Reclassified predicted risk(with hepcidin)				% (N) of subjects reclassified		
	<5%	5 to <10%	10 to <15%	≥15%	Increased risk	Decreased risk	Net correctly reclassified (%)
All-cause mortality							
Dead patients (87)							
<5%	18	9	7	1	42.5	11.4	31.1
5 to <10%	7	14	9	4	(37)	(10)	
10 to <15%	0	2	2	5			
≥15%	0	1	0	8			
Survival patients (672)							
<5%	451	63	8	1	16.7	7.14	-9.56
5 to <10%	33	48	16	9	(112)	(48)	
10 to <15%	2	5	6	15			
≥15%	0	3	5	7			
NRI (95% CI)							21.5 (10.5-35.7)
							*P*<0.001
Cardiovascular mortality							
Dead patients (66)							
<5%	14	11	3	0	40.9	10.6	30.3
5 to <10%	2	10	4	2	(27)	(7)	
10 to <15%	1	1	2	7			
≥15%	0	0	3	6			
Survival patients(693)							
<5%	495	59	3	0	14.1	7.34	-6.76
5 to <10%	29	43	15	13	(98)	(51)	
10 to <15%	2	5	7	9			
≥15%	1	1	5	6			
NRI (95% CI)							23.5 (9.51-38.4)
							*P*<0.001

NRI, net reclassification improvement.

## DISCUSSION

The findings of this study demonstrate that circulating hepcidin levels were higher in ACS patients when compared with stable CAD patients. Furthermore, plasma hepcidin levels were positively associated with mortality risks in ACS patients, independent of conventional CVD risk factors, including CRP and ferritin. The addition of hepcidin to the fully multivariable-adjusted models significantly improved the discrimination for all-cause and CVD mortality in ACS patients.

Several previous studies have reported that hepcidin concentrations were positively related to cardiovascular risks. In hemodialysis patients, hepcidin levels were positively associated with arterial stiffness, fatal and nonfatal cardiovascular events [21, 22]. Additionally, among the metabolic syndrome population, community population, and in non-alcoholic fatty liver disease patients, hepcidin concentrations were found to be an independent predictor of the presence of carotid plaques [18, 23, 24]. Importantly, we firstly demonstrated that plasma hepcidin levels are associated with the mortality risk of ACS patients. These findings suggest that plasma hepcidin could be considered a potential biomarker in assessing outcomes of ACS patients.

Elevated hepcidin levels in CAD patients could be explained by several factors. It has been recognized that serum ferritin levels as iron stores are mobilized and distributed-within the cells or other organs of body [25]. The higher serum ferritin concentrations among CAD patients have been well studied in many previous studies [26–28]. Furthermore, several studies revealed the positive association between the serum ferritin and hepcidin in healthy humans, even in CHD patients [29–31]. Consistently, we demonstrated a strong correlation between hepcidin and ferritin levels. Additionally, higher serum hepcidin modulation may contribute to the development of uremic accelerated atherosclerosis (UAAS) in CHD patients [31]. It has been assumed that the elevated serum hepcidin in chronic including CHD patients were probably due to the feedback responses resulting from elevated systemic iron indicated by elevated ferritin [32]. Furthermore, increased inflammatory stimulus is also a possible explanation of the elevated hepcidin levels. Variable degrees of inflammation characterize several common human disorders, such as atherosclerosis, obesity, diabetes and metabolic syndrome. At this regards, IL-6, as an important inflammatory cytokine, has been extensively studied. IL-6 stimulates hepcidin production, causes iron sequestration in macrophage stores and decreases tissue iron availability [33, 34]. Also, a study reported that circulating CRP was positively correlated with hepcidin in CHD patients with diabetic nephropathy [31]. Consistently, elevated hepcidin levels were positively correlated with CRP both in ACS and stable CAD patients in present study. However, there is no significant association between hepcidin and IL-6 in ACS patients, and the association in stable CAD patients was quite weak. This phenomenon needs to be further studied in large subject cohorts. Nevertheless, the inflammatory status of chronic disease patients might contribute partially to accelerate the increment of hepcidin levels.

Regarding the mechanism by which elevated hepcidin increases the mortality risk of ACS, it has been proposed that hepcidin increases iron deposition in macrophages within atherosclerotic plaques with subsequent increased lipid peroxidation and progression to foam cells [35]. Additionally, further evidence from recent *in-vitro* and *in-vivo* mice studies supports the hypothesis that suppression of hepatic hepcidin production resulted in reduced macrophage intracellular iron content, which increased the efflux capacity of cholesterol and thereby decreased the formation of foam cells and atherosclerosis [36]. Several studies have indicated that hepcidin intracellularly trapped iron in macrophages, increasing reactive oxygen species resulting in lipids peroxidation and decreased cholesterol efflux, which contributed to the plaque destabilization [20, 37]. The findings of our study further confirm that elevated hepcidin levels might accelerate the development of plaque destabilization.

In our study, both the C index and NRI demonstrated that hepcidin appeared to be a strong predictor of risk of mortality in ACS patients, independent of traditional cardiometabolic risk factors and inflammation markers. In ACS patients, cells in the myocardium or other tissues may suffer from low oxygen tension due to reduced blood supply, which may partly contribute to the increased hepcidin levels. On the other hand, it is possible that plaques are crushed or become vulnerable, which releases a large amount of hepcidin into circulating system in the ACS stage. Therefore, in the first few months following ACS, circulating hepcidin levels represent the iron excessive degree or ROS stress in arterial walls aggravating the severity of ACS and strongly predict future unfavorable cardiovascular events. As steeply high hepcidin following ACS state may gradually decline due to the acute phase protein property, and one may doubt whether transient changes are amenable to clinical intervention. However, Dutta et al demonstrated that acute events accelerated atherosclerosis and the influence lasted for months [38], in which the readily change of iron parameters might play a role. A study that measured the hepcidin level of 12 patients with AMI found that increased hepcidin levels remained high through day 7 [39]. The findings of these studies suggest that hepcidin stayed at abnormally high levels during the first few weeks after ACS and were timely therapy was useful.

The strengths of our study included the large sample size of CAD patients with different disease status, long follow-up time, and detection of hepcidin levels in CAD patients for the first time. Several potential limitations of our study deserve consideration. Firstly, we used a single baseline measurement of iron parameters; thus, we could not evaluate the possible effects of changes over time. Secondly, banked bio-samples were used for hepcidin detection, but any sample degradation should be random, and any resultant misclassification should only bias toward the null hypothesis.

## MATERIALS AND METHODS

A total of 1980 participants were recruited from the Guangdong Coronary Artery Disease Cohort between October 2008 and December 2011. Details of this cohort have been described before [40, 41]. The inclusion criteria were age ≥ 40 years old at baseline survey, a history or newly diagnosed of CAD (ICD-10 codes I20-I25) [42, 43], and residence in the Guangdong Province for more than 5 years. Patients with severe liver and/or kidney failure, autoimmune disease or thyroid disorders were excluded in the cohort. The additional exclusion criteria in the present study were as follows: 1) less than 50% coronary stenosis by CAG reports or not conducted CAG (n=489); 2) premenopausal women (n=25); 3) tumor disease patients (n=40); 4) anemia and/or iron deficiency treatment or using iron supplements within 3 months preceding the recruitment (n=27); and 5) inadequate blood samples for iron parameters detection (n=114). The final study sample was comprised of 1285 patients (759 patients with ACS and 526 patients with stable CAD) (Supplementary Figure 1). The ACS group consisted of subjects with high-risk unstable angina, ST or non-ST-elevated MI, and was defined as any vessel with ≥ 50% stenosis in the setting of elevated cardiac enzymes (creatinine kinase [CK] and CK-MB, troponin I, or troponin T) and/or dynamic electrocardiogram changes [44]. Chronic CAD was defined as ≥ 1 vessel with ≥ 50% stenosis and no acute coronary event more than 3 months [4]. Diagnosis of ACS and stable CAD was based on the physician’s judgment according to the above criteria, which was further confirmed by two cardiologists independently. The study was approved by the Sun Yat-sen University ethics committee; the protocol was consistent with the principles of the current revision of the Helsinki Declaration, and all participants provided informed consent.

General information such as examination and birth date, gender, education level, leisure-time physical activity, smoking, alcohol consumption, and medication history were conducted by an in-person interview during the admission process for each patient by trained research nurses. Hypertension was defined as systolic blood pressure ≥ 140 mmHg and/or diastolic blood pressure ≥ 90 mmHg and/or had a history of hypertension. Diabetes was defined as fasting plasma glucose level ≥ 7.0 mmol/L and/or had a history of diabetes. Venous blood samples were taken in the morning following at least 12 hours of fasting. After centrifuging, the serum and plasma were separated and stored at -80°C until further laboratory measurements were made. Treatment information about CAD included percutaneous coronary intervention and coronary artery bypass graft.

Plasma hepcidin-25was measured using a commercially available enzyme-linked immunosorbent assay (DRG international, Inc., USA (EIA-5782)). The minimum detectable dose (sensitivity) of hepcidin is typically less than 0.153ng/mL. No significant cross-reactivity or interference between hepcidin and analogues was observed. Intra- and inter-assay CV were 5.6% and 7.5%, respectively, which were measured in our own study. Plasma ferritin was determined using an immunoassay based on electrochemiluminescence with the Architect i4000 system (ABBOTT 65205 Wiesbaden, Germany). Serum iron and unsaturated iron-binding capacity were measured colorimetrically (Alpkem RFA analyzer, Alpkem Corporation, Clackamas, Oregon). Transferrin saturation was calculated as 100 × serum iron/(iron+ unsaturated iron-binding capacity). Serum soluble transferrin receptor was measured using immunonephelometry (Roche Diagnostics). The serum CRP and Interleukin-6 were assessed using Human FlowCytomix (Simplex BMS8288FF and BMS8213FF, eBioscience, USA) on the BD FACSCalibur instruments. All other biomarker tests were performed using standard techniques.

Follow-up data were obtained by a yearly review in recruiting hospitals, repeated telephone contacts with participants and/or their family members, and the death registry of the Guangdong Provincial Centers for Disease Control and Prevention. The follow-up surveys were conducted until the end of July 2014. The duration of follow-up was calculated from the recruitment date to July 31st, 2014 or to the patients’ death date. The average follow-up was 4.1 years. The ICD-10 codes were used to code the cause of death, and the ICD-10 codes I00-I99 were classified as CVD deaths.

Baseline differences in risk factors (continuous variables) between ACS and stable CAD groups were tested by ANCOVA after adjustment for age and gender. A Chi-squared test was used for categorical variables comparison. Correlation coefficients between markers were analyzed using Spearman’s test. The Cox proportional hazards model was used to estimate the association between hepcidin levels and the risk of all-cause and CVD mortality. Since hepcidin was not normally distributed, plasma hepcidin levels were log-transformed and then classified into tertiles (<33.3% [reference group], 33.3 to <66.7%, and ≥66.7%). The proportional hazards assumption in the Cox model was assessed with graphical methods and with models including time-by-covariate interactions [45]. In general, all proportionality assumptions were appropriate. Multivariable models were adjusted for age, gender, smoke, alcohol use, BMI, systolic blood pressure, low-density lipoprotein cholesterol, history of diabetes, history of heart failure, coronary artery disease severity, type, treatment, use of lipid-lowering, anti-hypertensive, glucose-lowering drugs, CRP, and ferritin. We calculated power at a sample size of 1285 and a Cox regression of the log hazard ratio on a covariate with a standard deviation of 0.38 based on a sample of 1285 observations achieves 79% power at a 0.05 significance level to detect a regression coefficient equal to 0.6 [46]. We computed the C index associated with the risk-estimation model based on all the classic risk factors listed above and the C index associated with the model based on a combination of the classic risk factors and hepcidin. The discriminative ability of the models including and excluding hepcidin was tested with the use of C index improvement. NRI was further used to assess the contribution of hepcidin [47]. We stratified patients into four risk categories (<5%, 5 to <10%, 10 to <15%, and ≥15%) based on the clinical variables. Statistical significance was considered to be *P*<0.05 (two sided). All statistical analyses were performed with PASW for Windows, version 20.0 (IBM SPSS Inc, Chicago, III), SAS for windows, version 9.4 (SAS Institute, Cary, NC), R for windows, version 2.12.1, and PASS, version 11 (NCSS, LLC).

## CONCLUSIONS

In conclusion, circulating hepcidin was positively associated with the risk of CVD and all-cause mortality in ACS patients. These findings indicated that hepcidin is considered as a non-invasive biomarker of prognosis in ACS patients.

## SUPPLEMENTARY MATERIALS FIGURE AND TABLE



## Abbreviation

ACS: acute coronary syndrome
BMI: body mass index
CAD: coronary artery disease
CAG: coronary angiography
CI: confidence intervals
CK: creatinine kinase
CRP: C-reactive protein
CVD: cardiovascular disease
HR: hazard ratio
ICD: international Classification of Diseases
MI: myocardial infarction
NRI: net reclassification improvement
UAAS: uremic accelerated atherosclerosis
