# Supplementary material for: Associations of plasma hepcidin with mortality risk in patients with coronary artery disease
Source: Oncotarget. 2017 Nov 27;8(65):109497–508. doi: 10.18632/oncotarget.22722 (PMC5752537; doi:10.18632/oncotarget.22722)
Supplement: Supplementary file 1 [file oncotarget-08-109497-s001.pdf]

Associations of plasma hepcidin with mortality risk in patients with coronary artery disease

SUPPLEMENTARY MATERIALS

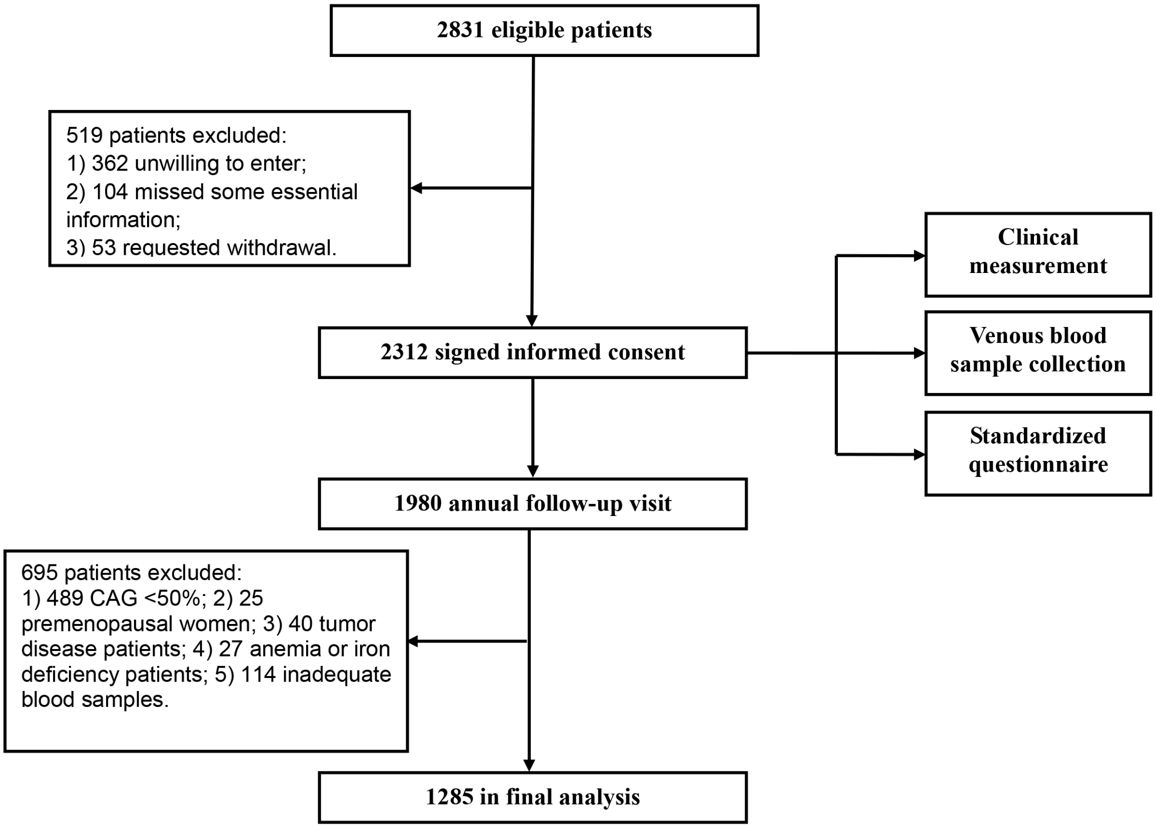

Supplementary Figure 1: Flow chart illustrating the recruitment of the patients for the study.

Supplementary Table 1: Correlation coefficients between markers

	IL-6	Ferritin	Hepcidin
Total Samples			
C-reactive protein	0.293*	0.278*	0.302*
Interleukin-6		0.131*	0.121*
Ferritin			0.873*
Acute coronary syndrome			
C-reactive protein	0.335*	0.205*	0.255*
Interleukin-6		0.094*	0.096
Ferritin			0.845*
Stable coronary artery disease			
C-reactive protein	0.229*	0.211*	0.275*
Interleukin-6		0.128*	0.131*
Ferritin			0.831*

* $P < 0.05$.
